# Delayed Diagnosis of Mild GLUT1 Deficiency Syndrome Caused by an Apparently De Novo SLC2A1 p.(Phe445del) Variant in a Child with a History of Severe Neonatal Hyperkalemia

**DOI:** 10.3390/children13070883

**Published:** 2026-06-30

**Authors:** Simona Ivančan, Maruša Debeljak, Tanja Loboda, Štefan Grosek

**Affiliations:** 1 Department of Pediatric Hematology and Oncology, University Children’s Hospital Ljubljana, University Medical Centre Ljubljana, 1000 Ljubljana, Slovenia; 2 Department of Pediatrics, Faculty of Medicine, University of Ljubljana, 1000 Ljubljana, Sloveniastefan.grosek@kclj.si (Š.G.); 3 Clinical Institute of Medical Genetics, University Children’s Hospital Ljubljana, University Medical Centre Ljubljana, 1000 Ljubljana, Slovenia; 4 Institute of Cell biology, Faculty of Medicine, University of Ljubljana, 1000 Ljubljana, Slovenia; 5 Department of Child, Adolescent and Developmental Neurology, University Children's Hospital Ljubljana, University Medical Centre, 1525 Ljubljana, Slovenia; tanja.loboda@kclj.si; 6 Neonatology Section, Department of Perinatology, Division of Gynaecology and Obstetrics, University Medical Centre Ljubljana, 1000 Ljubljana, Slovenia; 7 Department of Medical Ethics, Faculty of Medicine, University of Ljubljana, 1000 Ljubljana, Slovenia

**Keywords:** GLUT1 deficiency syndrome, *SLC2A1*, whole-exome sequencing, apparently de novo variant, neurocognitive phenotype, ADHD, hyperkalemia

## Abstract

**Highlights:**

A boy with severe transient neonatal hyperkalemia and bilateral congenital cataracts was later diagnosed with mild GLUT1 deficiency syndrome.

- Whole-exome sequencing identified an apparently de novo *SLC2A1* in-frame deletion despite normal brain MRI and EEG findings.
- Mild GLUT1 deficiency syndrome may remain unrecognized when symptoms are subtle, intermittent, and nonspecific. Genomic testing should be considered in children with unexplained neurological or neurocognitive symptoms after unrevealing routine investigations.

**Abstract:**

**Background/Objectives:** Glucose transporter type 1 deficiency syndrome (GLUT1DS) is a rare neurometabolic disorder with an expanding clinical spectrum, including mild and non-classical presentations. We report a boy with severe transient neonatal hyperkalemia, bilateral congenital cataracts, and later subtle neurological and neurocognitive symptoms, in whom genomic testing supported the diagnosis of mild GLUT1DS. **Methods:** This single-patient case report describes clinical follow-up from birth to nine years of age, including neurological, metabolic, neuropsychological, imaging, and genetic investigations. Whole-exome sequencing using next-generation sequencing technology was performed. **Results:** The patient required intensive care immediately after birth because of severe transient hyperkalemia of unclear etiology. Bilateral congenital cataracts were surgically corrected during infancy. Later, he developed two brief seizure episodes, reduced exercise tolerance, episodic fatigue, attentional difficulties, motor restlessness, and mild graphomotor impairment. Neuropsychological assessment showed overall average intellectual functioning, below-average verbal abilities, low-average non-verbal abilities, and attention-deficit/hyperactivity disorder. Repeated metabolic investigations, electroencephalography, and brain magnetic resonance imaging were unrevealing. Whole-exome sequencing identified an apparently de novo heterozygous *SLC2A1* variant, NM_006516.4.1333_1335del, p.(Phe445del), supporting the diagnosis of mild GLUT1DS. Because of the mild phenotype and preserved everyday functioning, ketogenic diet therapy was not initiated. **Conclusions:** This case highlights the diagnostic challenges of mild GLUT1DS and the value of genomic testing in children with unexplained neurological or neurocognitive symptoms despite normal routine investigations. Although neonatal hyperkalemia and GLUT1DS coexisted in this patient, current evidence is insufficient to establish a causal relationship.

## 1. Introduction

Glucose transporter type 1 deficiency syndrome (GLUT1DS) is a rare neurometabolic disorder caused by impaired glucose transport across the blood–brain barrier due to pathogenic variants in the *SLC2A1* gene [1,2]. The resulting reduction in cerebral glucose availability leads to chronic neuroglycopenia, particularly affecting the developing brain [1,2]. Classically, GLUT1DS presents with early-onset epilepsy, developmental delay, acquired microcephaly, and movement disorders [1,3,4]. However, the recognized clinical spectrum has expanded considerably, reflecting improved awareness of milder and non-classical presentations [3,4,5].

In addition to the classical phenotype, patients may present with paroxysmal exertion-induced dyskinesia, exercise intolerance, attentional fluctuations, episodic fatigue, learning difficulties, or infrequent seizures [3,4,5]. Such presentations may occur in the absence of structural abnormalities on brain magnetic resonance imaging (MRI) or epileptiform activity on electroencephalography (EEG), further complicating recognition [4,6]. Consequently, GLUT1DS remains underdiagnosed, particularly in patients with subtle, intermittent, or slowly evolving symptoms [5,7].

Traditionally, the diagnosis of GLUT1DS has relied on demonstration of hypoglycorrhachia, defined as reduced cerebrospinal fluid (CSF) glucose in the context of normoglycemia [1,2]. However, the increasing availability of next-generation sequencing (NGS), including whole-exome sequencing (WES), has significantly transformed the diagnostic approach [8,9]. Genomic testing may identify disease-associated *SLC2A1* variants in patients with atypical clinical features or incomplete biochemical evaluation, thereby reducing diagnostic delay and expanding the recognized phenotypic spectrum [3,8,9]. Variant interpretation should follow standardized ACMG/AMP recommendations and integrate population data, predicted molecular effect, segregation, and genotype–phenotype correlation [10].

Neonatal metabolic disturbances are not typical manifestations of GLUT1DS and may obscure later recognition of an underlying neurometabolic disorder [1,2]. We report a long-term follow-up of a boy previously described with severe neonatal hyperkalemia [11], whose later subtle neurocognitive and neurological symptoms led to the diagnosis of mild GLUT1DS caused by an apparently de novo *SLC2A1* p.(Phe445del) variant. The principal educational value of this case is not a proven causal link between hyperkalemia and GLUT1DS, but rather the prolonged diagnostic delay in a genetically confirmed mild GLUT1DS phenotype.

## 2. Case Presentation

The patient was born at term after an uncomplicated pregnancy and delivery. Immediately after birth, severe hyperkalemia was detected during routine laboratory evaluation, with critically elevated serum potassium requiring urgent transfer to the neonatal intensive care unit. Initial management included rapid stabilization using standard anti-hyperkalemic measures. Intravenous calcium gluconate was administered for cardiac membrane stabilization, followed by insulin–glucose infusion to promote intracellular potassium shift. Careful fluid and electrolyte management was maintained, and continuous cardiac monitoring was implemented because of the risk of life-threatening arrhythmias. The patient responded well, with gradual normalization of potassium levels over several hours.

Extensive diagnostic evaluation during the neonatal period did not reveal an underlying renal, endocrine, or metabolic cause. Renal function was normal, and no evidence of adrenal insufficiency or inborn error of metabolism was identified. The episode was therefore classified as severe transient neonatal hyperkalemia of unclear etiology, as previously reported [11]. Following stabilization, early neurological examinations and developmental milestones were unremarkable.

Congenital bilateral cataracts were diagnosed and surgically treated during the first year of life, with residual farsightedness. During the first 18 months of life, persistent reticulocytosis was noted. Extensive hematologic evaluation excluded hemolytic and red blood cell disorders, and no hereditary anemia was identified, including on later WES analysis. At two years of age, the patient experienced a brief generalized seizure without focal features. A second episode occurred at five years of age during febrile illness and physical exertion. Anticonvulsive therapy was not initiated.

From approximately five years onward, the parents reported reduced exercise tolerance, episodic fatigue, fluctuating attention, and increasing difficulties with sustained concentration. Cardiologic, endocrinologic, metabolic, and hematologic investigations remained within normal limits. No endocrine or systemic metabolic cause was identified, prompting reconsideration of an underlying neurometabolic disorder.

Formal neuropsychological assessment demonstrated overall average intellectual functioning. Verbal abilities were below average, whereas non-verbal intellectual abilities were within the low-average range. Mild impairment of fine graphomotor skills was documented. Motor restlessness and attentional difficulties were present, and the patient fulfilled diagnostic criteria for attention-deficit/hyperactivity disorder (ADHD). Despite these findings, academic performance remained age-appropriate and no significant cognitive decline was observed. A summary of the clinical course and key findings is presented in Table 1.

### 2.1. Diagnostic Evaluation

Given the combination of transient seizures, exertional fatigue, attentional variability, and subtle neuropsychological findings, a comprehensive reassessment was undertaken. Repeated EEG recordings were normal, and brain MRI showed no structural or signal abnormalities. CSF analysis was not performed. Lumbar puncture was not pursued because the patient was clinically stable, neuroimaging and EEG were normal, the phenotype was mild, and advanced genomic testing was available and considered diagnostically appropriate in the multidisciplinary evaluation.

Whole-exome sequencing was performed using NGS technology at the Clinical Institute of Medical Genetics, University Children’s Hospital Ljubljana. More than 95% of coding regions of the analyzed genes achieved adequate sequencing quality. Analysis identified a heterozygous in-frame deletion in exon 10 of *SLC2A1* (NM_006516.4:c.1333_1335del, p.(Phe445del)). The variant was absent from population databases, including gnomAD, and in silico prediction tools supported a deleterious effect (CADD score 26). According to ACMG/AMP recommendations, the variant fulfilled PM2, PM4, and PP3 criteria and was initially classified as likely pathogenic [10]. Subsequent parental testing demonstrated absence of the variant in both parents, supporting an apparently de novo occurrence. In the context of a compatible clinical phenotype (PP4), the molecular findings supported the diagnosis of mild GLUT1DS.

### 2.2. Treatment and Follow-Up

Following diagnosis, ketogenic diet therapy was discussed but was not recommended because of the patient’s mild clinical phenotype, infrequent seizure history, absence of a significant movement disorder, preserved everyday functioning, and overall favorable clinical course. The ketogenic diet remains the standard disease-specific treatment for GLUT1DS, particularly in patients with epilepsy or more severe phenotypes [1,2,12]. However, treatment decisions may require individualized consideration of symptom burden, tolerability, and expected benefit.

Empirical supplementation with B-complex vitamins and coenzyme Q10 was introduced and was well tolerated. During follow-up, the patient remained clinically stable, without further seizure episodes or neurological deterioration. However, neither B-complex supplementation nor coenzyme Q10 represents an established treatment for GLUT1DS, and their contribution to the observed clinical course cannot be determined. The possibility that the favorable course reflects the naturally mild phenotype of the disease cannot be excluded.

## 3. Discussion

This case illustrates the diagnostic complexity of mild GLUT1DS in a child with subtle and evolving neurological and neurocognitive manifestations. The early clinical course was dominated by severe neonatal hyperkalemia, a striking but currently unexplained event that was previously reported [11]. The later diagnosis of GLUT1DS was supported by an apparently de novo *SLC2A1* variant and a compatible mild phenotype rather than by a demonstrated mechanistic link with the neonatal electrolyte disturbance.

The patient’s neurological manifestations were subtle, intermittent, and slowly progressive, consisting of exertional fatigue, two brief seizure episodes, fluctuating attention, motor restlessness, and mild graphomotor impairment. This pattern is consistent with the expanding clinical spectrum of GLUT1DS, which includes non-classical presentations with exertional symptoms, paroxysmal neurological dysfunction, neurocognitive variability, and normal routine neuroimaging or EEG findings [3,4,5,6]. Normal EEG and MRI findings therefore do not exclude the diagnosis and may contribute to delayed recognition [4,6].

The formal neuropsychological profile further supported the diagnosis of a mild GLUT1DS phenotype. Although overall intellectual functioning remained within the average range, deficits in verbal abilities, attention regulation, graphomotor performance, and motor control were documented, together with ADHD. Such subtle neurocognitive manifestations may be under-recognized within the broader GLUT1DS spectrum and may contribute substantially to diagnostic delay, particularly when seizures are infrequent and structural investigations remain normal [3,5,7].

An important finding in this case was the apparently de novo occurrence of the identified *SLC2A1* variant. Neither parent carried the p.(Phe445del) variant, providing additional evidence supporting pathogenicity according to ACMG/AMP recommendations [10]. The combination of rarity in population databases, predicted deleterious effect, in-frame deletion in the encoded protein, segregation findings, and a compatible phenotype strengthens the diagnostic interpretation. Nevertheless, we acknowledge that published evidence specifically regarding p.(Phe445del) remains limited, and the classification should be understood in the context of the available clinical and segregation data.

The history of severe neonatal hyperkalemia remains an unusual and clinically important feature. Several *SLC2A1* variants have been associated with a cation-leak phenotype characterized by altered erythrocyte membrane permeability, hemolysis, pseudohyperkalemia or hyperkalemia, and stomatocytosis [13]. However, the p.(Phe445del) variant identified in our patient has not previously been reported to exhibit such functional properties. Furthermore, extensive hematologic evaluation did not identify chronic hemolysis, hereditary hemolytic anemia, or a red-cell membrane disorder. Therefore, while an association cannot be completely excluded, current evidence is insufficient to support a direct causal relationship between neonatal hyperkalemia and GLUT1DS in this patient.

Bilateral congenital cataracts represented another unusual finding. Cataracts are not recognized as a characteristic manifestation of GLUT1DS, and no established mechanistic relationship between impaired glucose transport due to *SLC2A1* haploinsufficiency and congenital lens opacification has been demonstrated. Their coexistence in this patient is therefore most likely coincidental. Similarly, the previous neonatal hyperkalemia should be interpreted as an unexplained coexisting finding rather than a proven manifestation of GLUT1DS.

The decision not to initiate ketogenic diet therapy should also be interpreted within the context of the patient’s mild phenotype. Ketogenic diet therapies provide ketone bodies as an alternative brain fuel and are the standard treatment for GLUT1DS, particularly in patients with epilepsy, movement disorders, or more disabling neurological manifestations [1,2,12]. In this patient, the limited seizure history, absence of significant movement disorder, preserved academic functioning, and stable clinical course led to an individualized decision against dietary treatment. Empirical vitamin and coenzyme Q10 supplementation was introduced, but no claim of disease-specific efficacy can be made.

The principal educational value of this case does not lie in a proven association between GLUT1DS and neonatal hyperkalemia, but rather in the prolonged diagnostic delay despite subtle neurological manifestations and a genetically supported neurometabolic disorder. The case emphasizes the importance of longitudinal reassessment and consideration of genomic testing in children with unexplained exertional fatigue, attentional variability, seizures, or neurocognitive symptoms, even when routine metabolic, EEG, and MRI investigations are unrevealing [8,9].

## 4. Conclusions

Mild forms of GLUT1DS may remain unrecognized for years because neurological manifestations can be subtle, intermittent, and nonspecific. This case highlights the importance of repeated clinical reassessment and demonstrates the increasing value of genomic testing in children with unexplained neurocognitive symptoms despite normal routine investigations. Severe neonatal hyperkalemia preceded the diagnosis by several years; however, current evidence does not support a direct pathophysiological relationship with GLUT1DS. The major lesson of this report is the potential for delayed recognition of mild GLUT1DS and the importance of considering this diagnosis even in patients with apparently nonspecific neurological symptoms.

## Figures and Tables

**Table 1:** Timeline of clinical course and key findings.

Age	Clinical Event	Investigations	Outcome
Birth	Severe hyperkalemia	Electrolytes, renal/endocrine/metabolic work-up	Stabilized; etiology unclear
Infancy	Bilateral congenital cataracts; persistent reticulocytosis	Ophthalmology; hematologic evaluation	Surgery; no hemolytic/red-cell disorder identified
2 years	Brief generalized seizure	EEG normal	No antiseizure therapy
5 years	Second brief seizure during febrile illness/exertion	EEG normal	No antiseizure therapy
5–9 years	Fatigue, reduced exercise tolerance, attention variability	Cardiologic, endocrine, metabolic and hematologic work-up negative	Persistent subtle symptoms
Follow-up	Formal neuropsychological assessment	Average intellectual functioning; below-average verbal abilities; low-average non-verbal abilities; graphomotor impairment; ADHD	Age-appropriate school performance
9 years	Genetic evaluation	WES/NGS: apparently de novo *SLC2A1* p.(Phe445del)	Mild GLUT1DS supported
After diagnosis	Management decision	Clinical monitoring	Ketogenic diet not recommended due to mild phenotype; empirical supplementation introduced

## Data Availability

The data presented in this study are not publicly available due to privacy and ethical restrictions involving identifiable clinical information. Additional anonymized data may be available from the corresponding author upon reasonable request.

## Abbreviations

The following abbreviations are used in this manuscript:

ADHD	attention-deficit/hyperactivity disorder
ACMG/AMP	American College of Medical Genetics and Genomics/Association for Molecular Pathology
CSF	cerebrospinal fluid
EEG	electroencephalography
GLUT1DS	glucose transporter type 1 deficiency syndrome
MRI	magnetic resonance imaging
NGS	next-generation sequencing
WES	whole-exome sequencing
